# Proteomic signatures of metronidazole-resistant *Trichomonas vaginalis* reveal novel proteins associated with drug resistance

**DOI:** 10.1186/s13071-020-04148-5

**Published:** 2020-06-01

**Authors:** Hsin-Chung Lin, Lichieh Julie Chu, Po-Jung Huang, Wei-Hung Cheng, Yu-Hsing Zheng, Ching-Yun Huang, Shu-Wen Hong, Lih-Chyang Chen, Hsin-An Lin, Jui-Yang Wang, Ruei-Min Chen, Wei-Ning Lin, Petrus Tang, Kuo-Yang Huang

**Affiliations:** 1 Division of Clinical Pathology, Department of Pathology, Tri-Service General Hospital, National Defense Medical Center, Taipei City, 114 Taiwan; 2 Molecular Medicine Research Center, Chang Gung University, Taoyuan City, 333 Taiwan; 3 Liver Research Center, Chang Gung Memorial Hospital, Linkou, Taoyuan City, 333 Taiwan; 4 Department of Biomedical Sciences, Chang Gung University, Taoyuan City, 333 Taiwan; 5 Genomic Medicine Core Laboratory, Chang Gung Memorial Hospital, Linkou, Taoyuan City, 333 Taiwan; 6 Molecular Regulation and Bioinformatics Laboratory, Department of Parasitology, College of Medicine, Chang Gung University, Taoyuan City, 333 Taiwan; 7 Graduate Institute of Pathology and Parasitology, National Defense Medical Center, Taipei City, 114 Taiwan; 8 Department of Medicine, Mackay Medical College, New Taipei City, 252 Taiwan; 9 Division of Infection, Department of Medicine, Tri-Service General Hospital SongShan Branch, Taipei City, 105 Taiwan; 10 Division of Family Medicine, Tri-Service General Hospital Songshan Branch, Taipei City, 105 Taiwan; 11 Graduate Institute of Biomedical and Pharmaceutical Science, Fu Jen Catholic University, New Taipei City, 242 Taiwan

**Keywords:** *Trichomonas vaginalis*, Metronidazole resistance, Proteome

## Abstract

**Background:**

Trichomoniasis is the most common non-viral sexually transmitted disease caused by the protozoan parasite *Trichomonas vaginalis*. Metronidazole (MTZ) is a widely used drug for the treatment of trichomoniasis; however, increased resistance of the parasite to MTZ has emerged as a highly problematic public health issue.

**Methods:**

We conducted iTRAQ-based analysis to profile the proteomes of MTZ-sensitive (MTZ-S) and MTZ-resistant (MTZ-R) parasites. STRING and gene set enrichment analysis (GESA) were utilized to explore the protein-protein interaction networks and enriched pathways of the differentially expressed proteins, respectively. Proteins potentially related to MTZ resistance were selected for functional validation.

**Results:**

A total of 3123 proteins were identified from the MTZ-S and MTZ-R proteomes in response to drug treatment. Among the identified proteins, 304 proteins were differentially expressed in the MTZ-R proteome, including 228 upregulated and 76 downregulated proteins. GSEA showed that the amino acid-related metabolism, including arginine, proline, alanine, aspartate, and glutamate are the most upregulated pathways in the MTZ-R proteome, whereas oxidative phosphorylation is the most downregulated pathway. Ten proteins categorized into the gene set of oxidative phosphorylation were ATP synthase subunit-related proteins. Drug resistance was further examined in MTZ-S parasites pretreated with the ATP synthase inhibitors oligomycin and bafilomycin A1, showing enhanced MTZ resistance and potential roles of ATP synthase in drug susceptibility.

**Conclusions:**

We provide novel insights into previously unidentified proteins associated with MTZ resistance, paving the way for future development of new drugs against MTZ-refractory trichomoniasis.
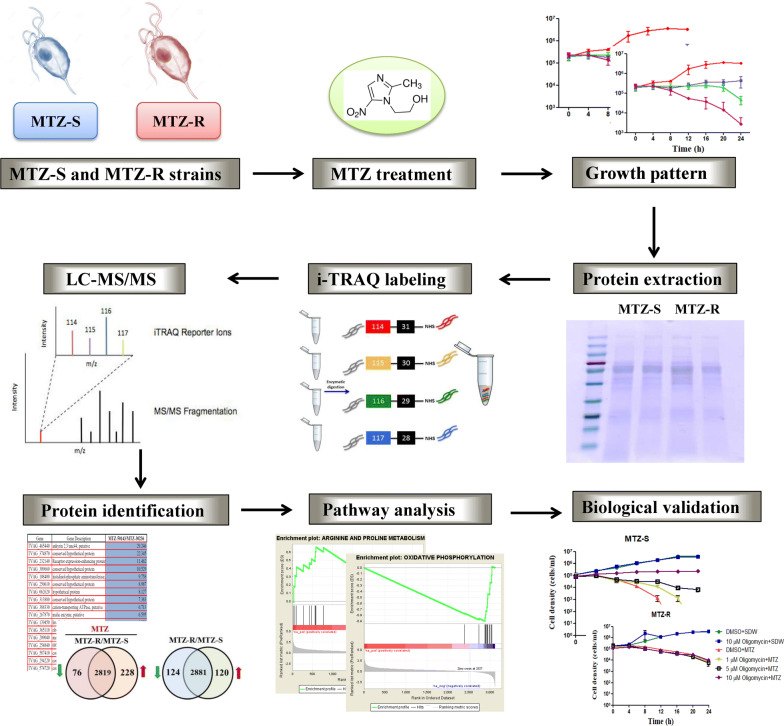

## Background

*Trichomonas vaginalis* is a unicellular, flagellated eukaryote that causes the most widespread non-viral sexually transmitted infection (STI), with more than 275 million cases reported annually worldwide [1]. Although men are often asymptomatic carriers of *T. vaginalis* infection, dysuria, discharge and an increased risk of prostate cancer have been reported [2]. Infected women develop vaginitis, urethritis, and cervicitis, potentially leading to adverse pregnancy outcomes, such as infertility, preterm delivery, and low-birth-weight infants [3]. Trichomoniasis has also been linked to an increased risk of human immunodeficiency virus (HIV) transmission [4] and cervical cancer [5]. Metronidazole (MTZ) and other 5-nitroimidazoles are the only two drugs currently approved by the FDA for the treatment of trichomoniasis, but MTZ-resistant (MTZ-R) strains are on the rise [6, 7]. It has been shown that 4.3% of *T. vaginalis* isolates display MTZ resistance in the USA [8]. Higher oral MTZ doses can sometimes cure refractory trichomoniasis but appear to be poorly tolerated [9]. Additionally, the teratogenic effect of MTZ on animal models is well documented [10–12]. Hence, it is essential to discover alternative chemotherapeutic agents against MTZ-R *T. vaginalis*.

Several mechanisms have been proposed to elucidate how pathogens resist to MTZ treatment, such as altered reduction efficiency [13], drug inactivation [14], reduced drug uptake [15], active drug efflux [16], and increased DNA damage repair [17]. Previous work showed that deficient pyruvate:ferredoxin oxidoreductase (PFOR) activity in *T. vaginalis* leads to low levels of anaerobic resistance to MTZ [18]. Another report indicated that MTZ-R trichomonads have decreased ferredoxin (Fd) levels [19]. A recent transcriptomic study in *Entamoeba histolytica* demonstrated that MTZ resistance is associated with specific transcriptional changes [20]. These findings suggest that various mechanisms are involved in MTZ resistance in different protozoan parasites. It is worthwhile to advance our knowledge of the mechanism of MTZ resistance in *T. vaginalis* by using multi-omics approaches, such as next-generation RNA sequencing (RNA-seq) or quantitative proteomic analysis, which is helpful to identify the novel players regulating MTZ resistance.

Although the draft genome sequence of *T. vaginalis* has been reported [21], the progress in studying the biology of trichomonads by using proteomic technologies is slow. The initial proteomic study utilized 2-dimentional gel electrophoresis (2-DE) combined with matrix-assisted laser desorption ionization time-of-flight (MALDI-TOF) mass spectrometric analysis to establish the proteome reference map of *T. vaginalis* [22]. Subsequent studies focused on the profiling of subcellular proteomes, such as the hydrogenosome [23] and surface proteomes [24]. Comparative proteomic analyses were conducted to identify the potentially important proteins involved in the adherence of trophozoites to fibronectin [25], iron availability [26], and the transformation of trophozoites to pseudocysts [27]. Additionally, differentially expressed proteins were identified from bovine and feline *Tritrichomonas foetus* genotypes, providing evidence of host-specific adaptation [28]. A recent comparative proteomic analysis of the membrane proteins has been carried out in another trichomonad *Trichomonas gallinae*, showing distinct proteins associated with virulence [29].

As an increased resistance to MTZ has emerged as a highly problematic public health issue in several protozoan infections, we aim to unveil the molecular events of MTZ resistance in *T. vaginalis* using the isobaric tags for relative and absolute quantitation (iTRAQ)-based quantitative proteomic approach. The findings of this study will advance our understanding of the mechanism of MTZ resistance in the amitochondriate organisms and pave the way for development of alternative drugs against MTZ-refractory trichomoniasis.

## Methods

### *Trichomonas vaginalis* strains and culture conditions

The *T. vaginalis* MTZ-S strain (ATCC 30236) and MTZ-R strain (ATCC 50143) were maintained in YIS medium [30], pH 5.8, containing 10% heat-inactivated horse serum and 1% glucose at 37 °C. Growth of the parasites was monitored by using trypan blue exclusion hemocytometer counts.

### MTZ susceptibility assay

To determine the concentration and incubation time of MTZ for proteomic analysis, the cell density of both MTZ-S and MTZ-R parasites was monitored every 4 h after treatment with different concentrations of MTZ (5, 10, 20 μM) (Sigma-Aldrich, Saint Louis, MO, USA) compared with that of the sterile distilled water (SDW)-treated control.

### Sample preparation and iTRAQ labeling

Whole cell lysates extracted from the MTZ-S and MTZ-R parasites treated with MTZ (20 μM for 8 h) or SDW were harvested and resuspended in 100 µl RapiGest SF surfactant (Waters Corporation, MA, USA). The protein concentrations in samples were determined using a BCA protein assay kit (Pierce, Thermo Fisher Scientific, Illinois, USA). The protein samples (10 µg) were separated with 10% sodium dodecyl sulfate polyacrylamide gel electrophoresis (SDS-PAGE) (Future Scientific Co., Taoyuan, Taiwan) followed by Coomassie blue staining using standard procedures. Each sample (20 µg) was first reduced with 5 mM tris-(2-carboxyethyl)-phosphine (TCEP; Sigma-Aldrich) at 60 °C for 30 min, followed by cysteine-blocking with 10 mM methyl methanethiosulfonate (MMTS; Sigma-Aldrich) at 25 °C for 30 min, and digested at 37 °C for 16 h by trypsin (1 µg; Promega, Madison, USA) in a solution containing 150 mM TEABC. The peptides were then labeled with iTRAQ reagent (ABSciex, CA, USA) according to the manufacturer’s protocol. After incubation at room temperature for 1 h, the six peptide mixtures were pooled, dried by vacuum centrifugation, and store at − 80 °C until use.

### LC-MS/MS analysis for protein identification

The dried peptide mixtures were reconstituted with 30% acetonitrile/0.1% formic acid for analysis using a LTQ-Orbitrap ELITE mass spectrometer (Thermo Fisher Scientific) as previously described [31]. The MS raw data files were analyzed by Proteome Discoverer software (version 1.4; Thermo Fisher Scientific) including the reporter ions quantifier node for iTRAQ quantification. The MS/MS spectra was searched against the TrichDB-33_TvaginalisG3 sequence database (97,471 entries) using the Mascot search engine (version 2.5; Matrix Science, London, UK). For peptide identification, 10 ppm mass tolerance was permitted for intact peptide masses, and 0.05 Da for HCD fragment ions with an allowance for two missed cleavages made from the trypsin or semi-trypsin digestion: oxidized methionine, acetyl (protein N-terminal), iTRAQ (N-terminal), and iTRAQ (lysine) as variable modifications; and Methylthio (cysteine) as the fixed modifications. The peptide-spectrum match (PSM) was then filtered based on high confidence and Mascot search engine rank 1 of peptide identification to ensure an overall false discovery rate below 0.01. Proteins with a single peptide hit were removed. The identified proteins with fold changes higher than 2-fold or lower than 0.5-fold in the MTZ-R proteome upon MTZ treatment were considered differentially expressed in this study.

### Proteomic data analysis

Differential protein expression analysis in the MTZ-R proteome in response to drug treatment compared with that of MTZ-S was performed using Gene Set Enrichment Analysis (GSEA) [32]. *Trichomonas vaginalis*-specific databases with functional annotation of all identified proteins based on the Kyoto Encyclopedia of Genes and Genomes (KEGG) [33] and Gene Ontology (GO) [34] were incorporated into the GSEA software as reference databases. Protein-protein interaction networks were analyzed by STRING [35]. The STRING database collects and integrates known and predicted protein-protein association data for a large number of organisms, including *T. vaginalis*. A protein identifier was inserted in STRING and the network view summarizes the network of predicted associations for a particular group of proteins.

### RNA extraction, cDNA synthesis and quantitative PCR (qPCR)

Total RNA was extracted from MTZ-S and MTZ-R parasites treated with MTZ (20 μM) or SDW for 8 h using the SV total RNA isolation system (Premega). Reverse transcription (RT) was carried out using the SuperScript^TM^III First-Strand synthesis system (Thermo Fisher Scientific). qPCR was performed as previously described with minor modifications [25]. Briefly, the qPCR was performed by using SYBR Green qPCR Master Mix (Bio-Rad, CA, USA) on a ViiA7 real-time PCR system (Thermo Fisher Scientific). 60S rRNA was used as an internal control for normalization of gene expression in all experimental groups. Primer pairs used in this study are listed in Additional file 1: Table S1.

### ATP synthase inhibition assay

To verify the possible role of ATP synthase in MTZ resistance, MTZ-S and MTZ-R trophozoites were pretreated with different concentrations of oligomycin (1 μM, 5μM and 10 μM) (Sigma-Aldrich, Saint Louis, MO, USA) or bafilomycin A1 (100 nM and 500 nM) (Sigma-Aldrich, Saint Louis, MO, USA) for 1 h, and the susceptibility to MTZ was monitored as compared to the DMSO-treated control.

### Statistical analysis

Quantitative data were expressed as mean ± SD of three independent experiments unless otherwise indicated. A Student’s t-test (two-tailed) was used to evaluate the significant differences between groups. *P* < 0.05 was considered statistically significant.

## Results

### The effect of MTZ on the growth of MTZ-S and MTZ-R *T. vaginalis*

**Fig. 1:**
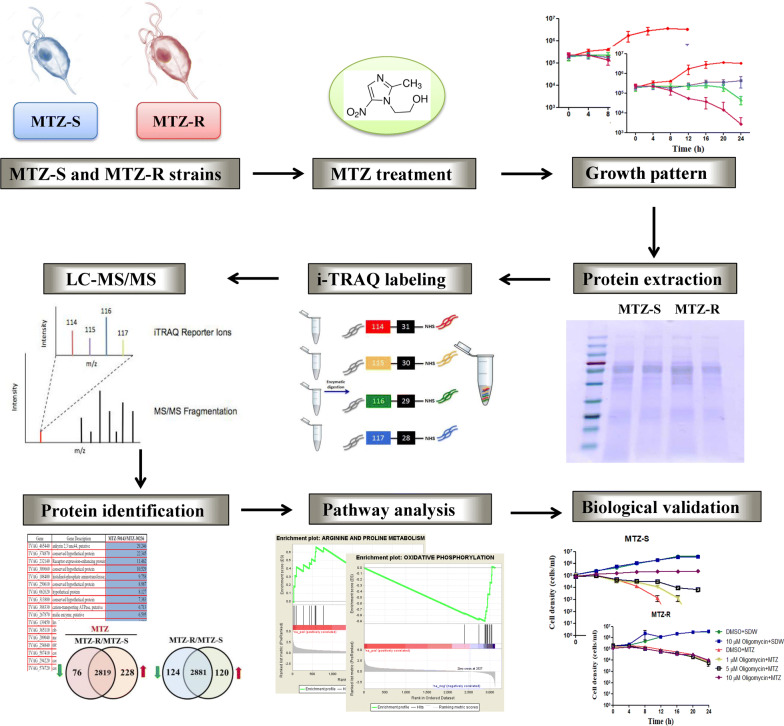
A schematic diagram of experimental workflow. To explore the underlying mechanism of MTZ resistance, we conducted quantitative proteomic analysis to profile global proteomes of MTZ-S and MTZ-R parasites. iTRAQ combined with LC-MS/MS analysis were performed to identify the differentially expressed proteins in the MTZ-R proteome. Functional annotations, protein-protein interaction networks and pathway enrichment analysis of the differentially expressed proteins were analyzed by GO, STRING and GESA, respectively. Proteins potentially associated with MTZ resistance were selected for experimental validation

**Fig. 2:**
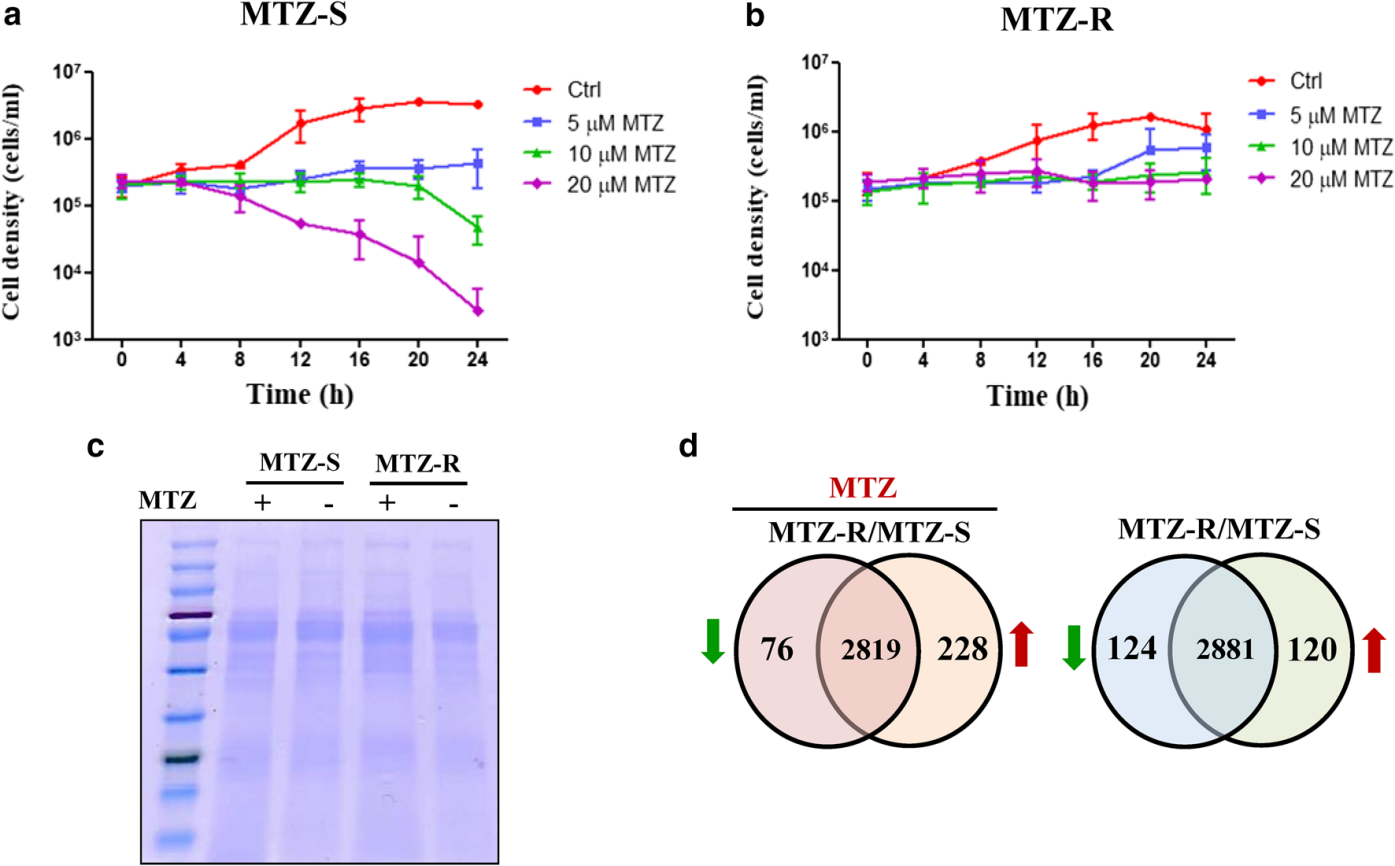
Identification of differentially expressed proteins between the MTZ-S and MTZ-R proteomes. To determine the concentration and incubation period of MTZ that could differentiate the growth patterns between MTZ-S (ATCC 30236) and MTZ-R (ATCC 50143) parasites, the cell density of trophozoites treated with different concentrations of MTZ was monitored for 24 h. The initial concentration of both MTZ-S and MTZ-R strains was 2 × 10^5^ cells/ml. MTZ-S trophozoites (**a**) and MTZ-R trophozoites (**b**) were treated with MTZ (5 μM, 10 μM or 20 μM) compared with the SDW-treated control (Ctrl). The cell density was monitored every 4 h by trypan blue exclusion assay. The growth curves were presented as mean ± SD of three independent experiments. **c** Whole cell lysates extracted from MTZ-S and MTZ-R parasites in the presence or absence of MTZ were separated with 10% SDS-PAGE followed by Coomassie blue staining. **d** A total of 3123 and 3125 proteins were identified in the paired MTZ-R and MTZ-S proteomes treated with or without MTZ, respectively. Among these identified proteins, 228 and 76 proteins were upregulated and downregulated, respectively, in the MTZ-R proteome treated with MTZ. Additionally, 120 and 124 proteins were upregulated and downregulated respectively, in the MTZ-R proteome without MTZ treatment

The workflow started from the extraction of proteins of MTZ-S and MTZ-R parasites treated with or without MTZ to quantitative proteomic analysis, followed by *in silico* pathway analysis and functional validation, is shown in Fig. 1. To determine the concentration and incubation period of MTZ that could differentiate the growth patterns between MTZ-S and MTZ-R parasites, the cell density of trophozoites treated with different concentrations of MTZ (5, 10 and 20 μM) was monitored for 24 h (Fig. 2a, b). Treatment with 5 μM MTZ maintained the survival of MTZ-S parasites at least within 24 h, whereas MTZ-R parasites can still replicate after 16 h of the same treatment. Treatment with 10 μM MTZ inhibited the growth of MTZ-R parasites and induced cell death of MTZ-S parasites after 20 h of treatment. The inhibitory effect of 20 μM MTZ treatment on the growth of MTZ-R parasites was similar to that of 10 μM MTZ treatment, but this dosage can lead to rapid cell death of MTZ-S parasites after 8 h of treatment. Since 8 h of 20 μM MTZ treatment was able to differentiate the growth patterns between MTZ-S and MTZ-R parasites, we used this condition for subsequent proteomic analysis.

### Quantitative proteomic analysis of MTZ-S and MTZ-R isolates

To identify the differentially expressed proteins associated with MTZ resistance, whole cell lysates extracted from MTZ-S and MTZ-R trophozoites treated with MTZ (20 μM for 8 h) were subjected to quantitative proteomic profiling using iTRAQ combined with LC-MS/MS analysis (Fig. 2c). Proteomic profiling of MTZ-S and MTZ-R trophozoites without drug treatment was also analyzed, representing strain-specific protein expression patterns. A total of 3123 and 3125 proteins were identified from both isolates treated with or without MTZ, respectively (Fig. 2d, Additional file 2: Table S2). Among the identified proteins, we obtained 304 differentially expressed proteins (9.7% of the identified MTZ-R/MTZ-S proteomes) in the MTZ-R proteome upon MTZ treatment, including 76 downregulated and 228 upregulated proteins. Additionally, we obtained 244 differentially expressed proteins (7.8% of the identified MTZ-R/MTZ-S proteomes) in the MTZ-R proteome without MTZ exposure, including 124 downregulated and 120 upregulated proteins. To validate the proteomic data, we determined the mRNA expression levels of 4 genes with 2-fold upregulation or downregulation in the MTZ-R proteome upon MTZ treatment (Additional file 1: Table S1). qPCR analysis confirmed the expression patterns of these transcripts, which were in agreement with the protein expression patterns (Additional file 3: Figure S1).

### Protein-protein interaction network analysis of the most differentially expressed proteins in the MTZ-R proteome upon MTZ treatment

**Fig. 3:**
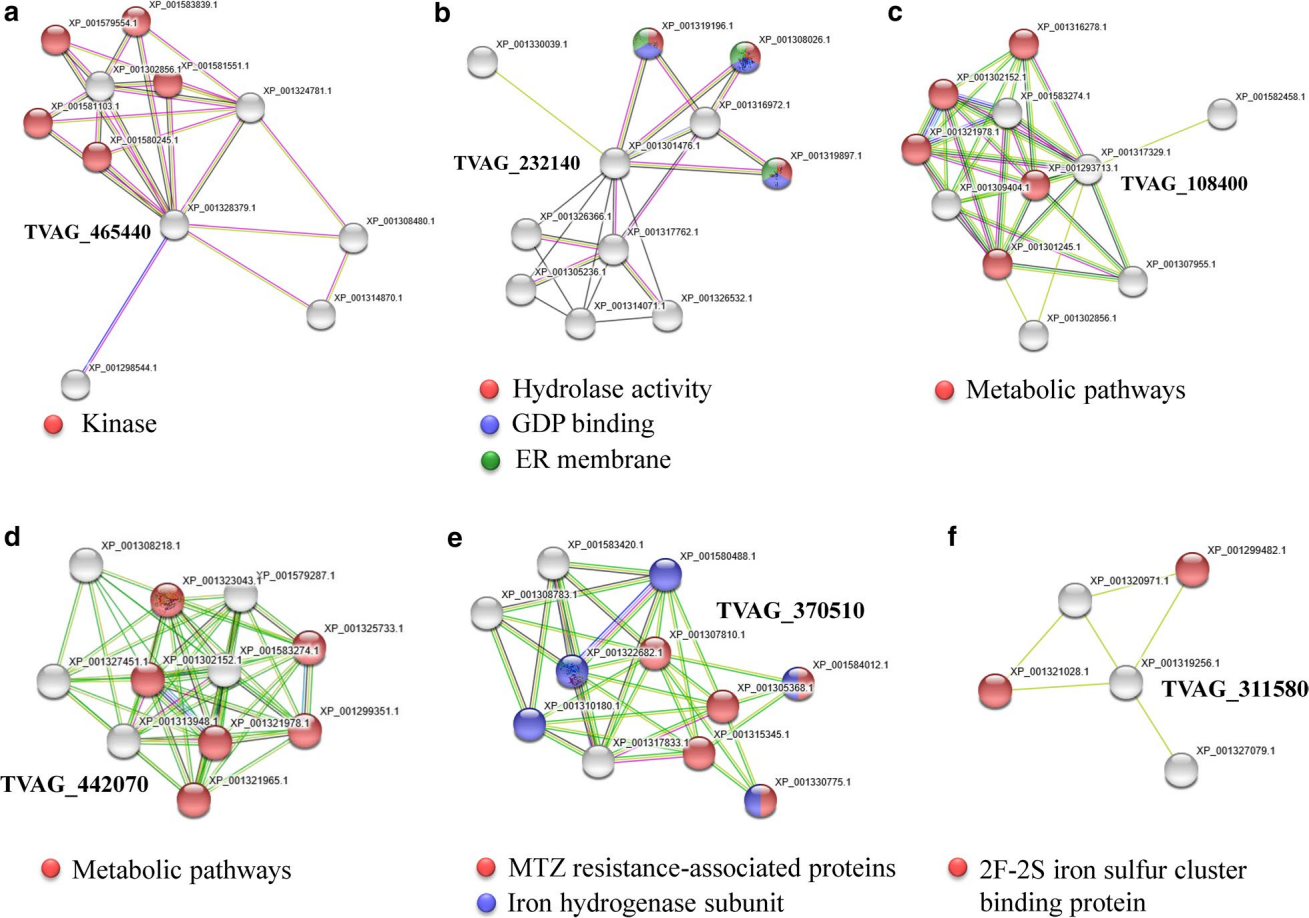
Network and enrichment analysis of the most differentially expressed proteins in the MTZ-R proteome upon MTZ treatment. Using STRING analysis, the protein-protein interaction networks were obtained upon entering the most upregulated proteins ankyrin 2,3/unc44 (TVAG_465440) (**a**), receptor expression-enhancing protein (TVAG_232140) (**b**) and histidinol-phosphate aminotransferase (TVAG_108400) (**c**), as well as the most downregulated proteins glucose kinase (TVAG_442070) (**d**), iron-sulfur flavoprotein (TVAG_370510) (**e**), and NAD(P)H dehydrogenase (TVAG_311580) (**f**) in the MTZ-R proteome in response to MTZ. As shown at the bottom of each panel, the enriched functions of the networks have been identified, and the corresponding protein nodes in the networks were automatically highlighted in colors

Among the 304 differentially expressed proteins between the MTZ-R and MTZ-S proteomes in response to MTZ treatment, 21 proteins (0.7% of the identified MTZ-R/MTZ-S proteomes) showed more than a 5-fold increase in MTZ-R parasites (Table 1), whereas 13 proteins (0.4% of the MTZ-R/MTZ-S proteomes) showed more than a 5-fold decrease in MTZ-R parasites (Table 2). The most upregulated proteins in MTZ-R parasites treated with MTZ were ankyrin 2,3/unc44 (TVAG_465440, 29.25-fold), receptor expression-enhancing protein (TVAG_232140, 11.46-fold), and histidinol-phosphate aminotransferase (TVAG_108400, 9.76-fold). On the other hand, the most downregulated proteins in MTZ-R parasites treated with MTZ were glucose kinase (TVAG_442070, 0.09-fold), iron-sulfur flavoprotein (TVAG_370510, 0.11-fold), and NAD(P)H dehydrogenase (TVAG_311580, 0.13-fold). Protein-protein interaction networks of these significantly dysregulated proteins in the MTZ-R proteome upon drug treatment were further constructed by STRING. The enrichment tests for functional associations have been incorporated in STRING, such as GO, KEGG, Pfam and InterPro. Ankyrin 2,3/unc44 was predicted to interact with several kinases (Fig. 3a). Receptor expression-enhancing protein was shown to be associated with a cluster of proteins with hydrolase activity and GDP-binding site on the ER membrane compartment (Fig. 3b). Histidinol-phosphate aminotransferase and glucose kinase were linked to metabolic pathways (Fig. 3c, d). It is noteworthy to mention that iron-sulfur flavoprotein and its interactive proteins with the iron hydrogenase subunit have also been identified to be downregulated in other MTZ-R *T. vaginalis* stains [36] (Fig. 3e). NAD(P)H dehydrogenase was associated with the proteins with 2F-2S iron sulfur cluster binding domain (Fig. 3f).

**Table 1:** The most upregulated proteins in the MTZ-R proteome in response to MTZ

Gene ID	Protein name	Ratio MTZ-R/MTZ-S
TVAG_465440	Ankyrin 2,3/unc44, putative	29.246
TVAG_374870	Conserved hypothetical protein	22.345
TVAG_232140	Receptor expression-enhancing protein, putative	11.462
TVAG_309660	Conserved hypothetical protein	10.529
TVAG_108400	Histidinol-phosphate aminotransferase, putative	9.758
TVAG_250610	Conserved hypothetical protein	8.987
TVAG_062620	Hypothetical protein	8.127
TVAG_313880	Conserved hypothetical protein	7.383
TVAG_388330	Cation-transporting ATPase, putative	6.713
TVAG_267870	Malic enzyme, putative	6.595
TVAG_139450	Involucrin, putative	6.300
TVAG_365110	Ribokinase, putative	6.225
TVAG_209040	Methyltransferase, putative	6.178
TVAG_254840	60S ribosomal protein L12, putative	6.146
TVAG_587410	Conserved hypothetical protein	5.871
TVAG_294220	Conserved hypothetical protein	5.655
TVAG_574720	Conserved hypothetical protein	5.525
TVAG_488940	Conserved hypothetical protein	5.440
TVAG_049830	Disulfide oxidoreductase, putative	5.439
TVAG_219180	Conserved hypothetical protein	5.118
TVAG_335530	Conserved hypothetical protein	5.028

**Table 2:** The most downregulated proteins in the MTZ-R proteome in response to MTZ

Gene ID	Protein name	Ratio MTZ-R/MTZ-S
TVAG_442070	Glucose kinase, putative	0.085
TVAG_004670	Conserved hypothetical protein	0.085
TVAG_370510	Iron-sulfur flavoprotein	0.111
TVAG_311580	NAD(P)H dehydrogenase, putative	0.127
TVAG_596850	Conserved hypothetical protein	0.133
TVAG_147370	Conserved hypothetical protein	0.137
TVAG_466240	Conserved hypothetical protein	0.154
TVAG_354010	Conserved hypothetical protein	0.155
TVAG_483090	40S ribosomal protein S2, putative	0.158
TVAG_270500	Arginine/serine-rich splicing factor, putative	0.165
TVAG_484570	Alkyl hydroperoxide reductase, subunit C, putative	0.186
TVAG_440200	Conserved hypothetical protein	0.189
TVAG_484130	Galactokinase, putative	0.199

### Differentially expressed proteins involved in the hydrogenosomal metabolism of the MTZ-R proteome upon MTZ treatment

**Fig. 4:**
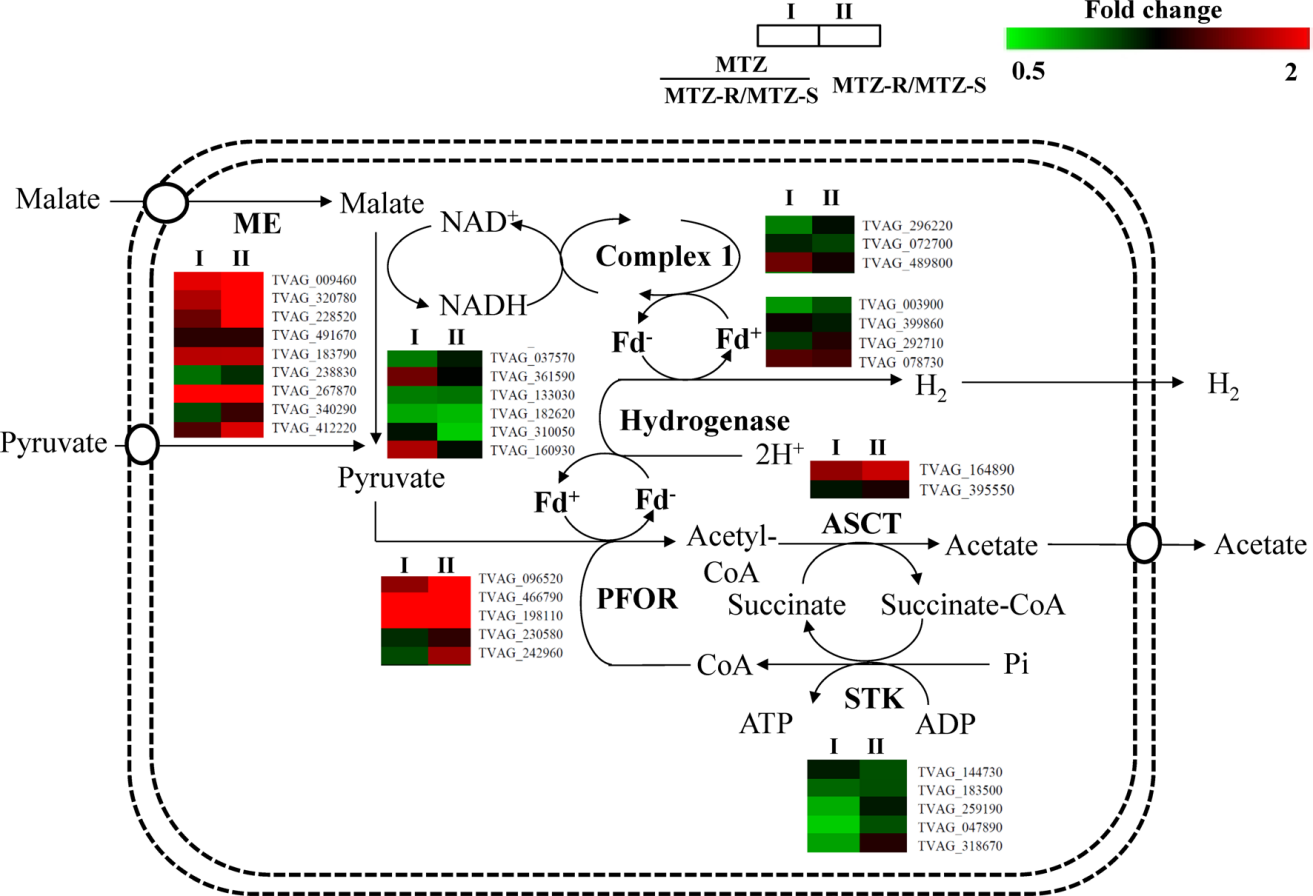
Differentially expressed proteins involved in the hydrogenosomal energy metabolism of the MTZ-R proteome. The protein expression changes between the MTZ-R and MTZ-S proteomes treated with MTZ (MTZ-MTZ-R/MTZ-S; I) or without MTZ (MTZ-R/MTZ-S; II) were presented as fold change. Proteins shown in red and green color indicate upregulation and downregulation, respectively. Complex I, also known as NADH dehydrogenase, is composed of two subunits (24 kDa subunit: TVAG_296220; 51 kDa subunit: TVAG_072700 and TVAG_489800). *Abbreviations*: ME, malic enzyme; Fd, ferredoxin; PFOR, pyruvate:ferredoxin oxidoreductase; ASCT, acetyl:succinate CoA-transferase; STK, succinate thiokinase

It has been shown that laboratory-generated MTZ resistance is associated with downregulation of specific hydrogenosomal enzymes that reduce MTZ, such as PFOR and Fd, but the clinical resistant strains showed no decrease in PFOR or Fd transcription [37, 38]. We thus analyzed the differentially expressed proteins involved in hydrogenosomal metabolism [21] between the MTZ-R and MTZ-S proteomes in the presence or absence of MTZ (Fig. 4, Additional file 4: Table S3). Two of five PFOR proteins (TVAG_230580, TVAG_242960) were downregulated in the MTZ-R proteome upon drug treatment. Among the four identified Fd proteins, Fd1 (TVAG_003900, 0.635-fold) was the most downregulated protein in the MTZ-proteome compared with that of MTZ-S in response to drug treatment. Particularly noteworthy is that all identified succinate thiokinase (STK) (TVAG_183500, TVAG_144730, TVAG_259190, TVAG_047890 and TVAG_318670), which catalyze ATP synthesis in the hydrogenosome, were significantly downregulated in the MTZ-R proteome in response to drug treatment. This suggests that the hydrogenosomal energy metabolism was suppressed in MTZ-R parasites upon drug treatment.

### GSEA revealed altered metabolic signatures in the MTZ-R proteome after drug treatment

**Fig. 5:**
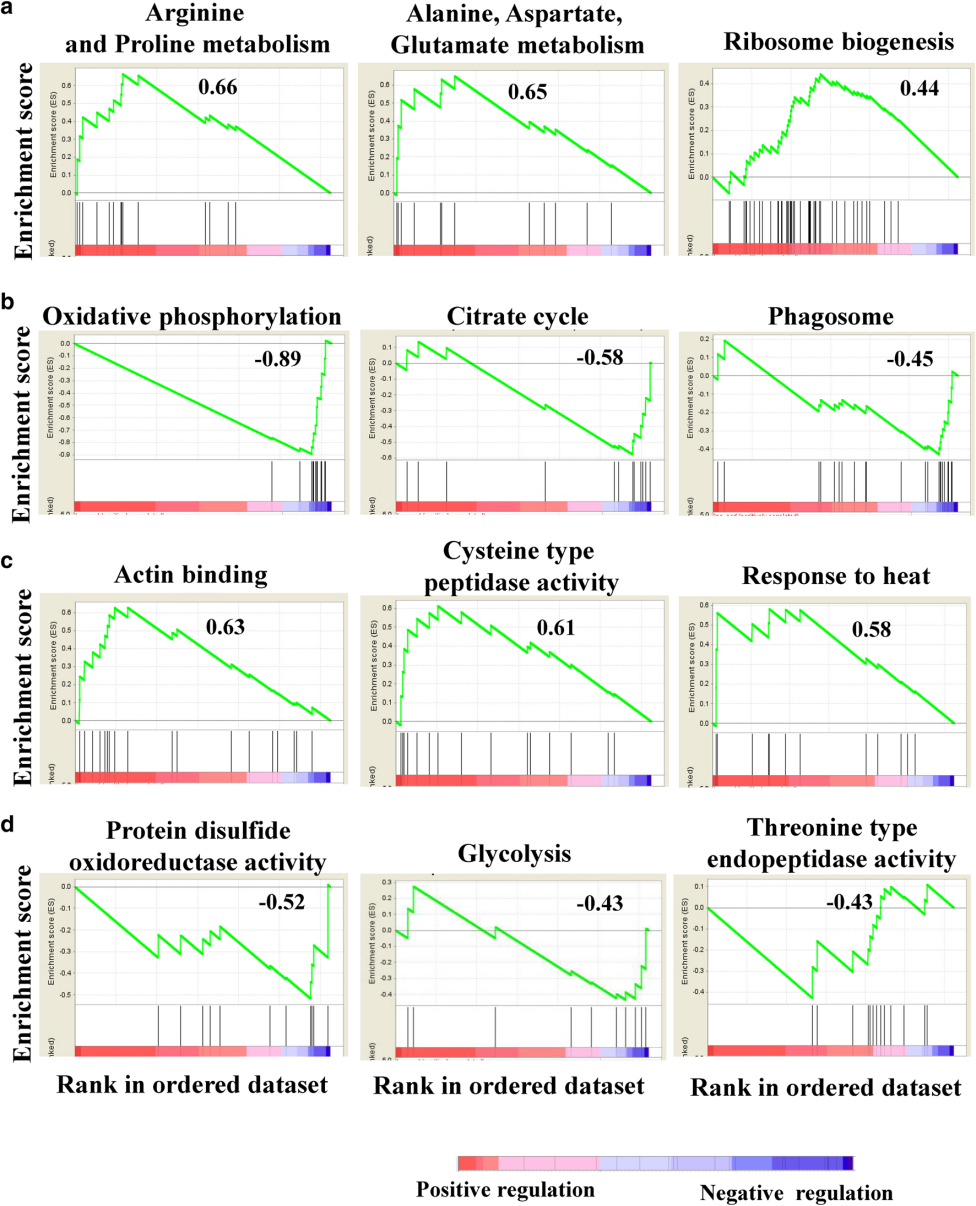
Enrichment analysis of differentially expressed proteins reveals enriched gene sets in the MTZ-R proteome upon MTZ treatment. A total of 3123 proteins ranked by fold change in protein expression (MTZ-R proteome compared with that of MTZ-S) were subjected to GSEA. Enrichment score (ES) reflects the degree of a gene set to be overrepresented at the top or bottom of a ranked list of genes. The positive ES and negative ES indicate upregulation and downregulation of a specific gene set, respectively. The enrichment plots using KEGG as the database presented the top 3 upregulated (**a**) or downregulated (**b**) pathways in the MTZ-R proteome upon MTZ treatment. The enrichment plots using GO as the database showed the top 3 enriched GO terms of the upregulated (**c**) or downregulated (**d**) gene sets in the MTZ-R proteome upon MTZ treatment

To globally assess the differences between the MTZ-R and MTZ-S proteomes upon drug treatment, we utilized Gene Set Enrichment Analysis (GSEA) to identify the enriched protein sets, both positive and negative regulation, according to KEGG (Additional file 5: Table S4, Additional file 6: Table S5) and GO (Additional file 7: Table S6, Additional file 8: Table S7) functional annotations. GSEA for KEGG pathway mapping showed that the most upregulated pathways in the MTZ-R proteome upon drug treatment were arginine and proline metabolism (enrichment score, ES = 0.66), alanine, aspartate, and glutamate metabolism (ES = 0.65), and ribosome biogenesis in eukaryotes (ES = 0.44) (Fig. 5a), whereas the most downregulated pathways were oxidative phosphorylation (ES = − 0.89), citrate cycle (ES = − 0.58), and phagosome (ES = − 0.45) (Fig. 5b). GSEA based on GO categories revealed that the most upregulated pathways in the MTZ-R proteome upon drug treatment were actin binding (ES = 0.63), cysteine type peptidase activity (ES = 0.61), and response to heat (ES = 0.58) (Fig. 5c), whereas the most downregulated protein sets belonged to protein disulfide oxidoreductase activity (ES = − 0.52), glycolysis (ES = − 0.43), and threonine type endopeptidase activity (ES = − 0.43) (Fig. 5d). Based on the GSEA data enriched from the MTZ-R proteome after drug treatment, it seems that there was a metabolic reprogramming toward reduced glycolysis but increased amino acid metabolism in MTZ-R parasites.

### Several aminotransferases were upregulated in the MTZ-R proteome upon MTZ exposure

We further analyzed the individual dysregulated proteins in these enriched protein sets. Specifically, several aminotransferases categorized into arginine, proline, aspartate, and glutamate metabolism, such as glucosamine-fructose-6-phosphate aminotransferase (TVAG_478320 and TVAG_011780), aspartate aminotransferase (TVAG_431780, TVAG_430210 and TVAG_419720), alanine aminotransferase (TVAG_088220), and glutamate dehydrogenase (GDH) (TVAG_025910) were upregulated and statistically enriched in the MTZ-R proteome after drug treatment. On the other hand, several proteins involved in glycolysis that link to hydrogenosomal energy metabolism, such as glyceraldehyde 3-phosphate dehydrogenase (TVAG_193800), phosphoglycerate mutase (TVAG_209020), and PFOR (TVAG_230580 and TVAG_242960), were downregulated in the MTZ-R proteome after drug treatment. These results suggest that specific reactions catalyzed by these aminotransferases are activated in MTZ-R parasites in response to MTZ; however, it requires further investigation whether this metabolic reprogramming is an outcome or a cause of MTZ resistance.

### Potential roles of ATP synthase subunit-related proteins in MTZ susceptibility

**Fig. 6:**
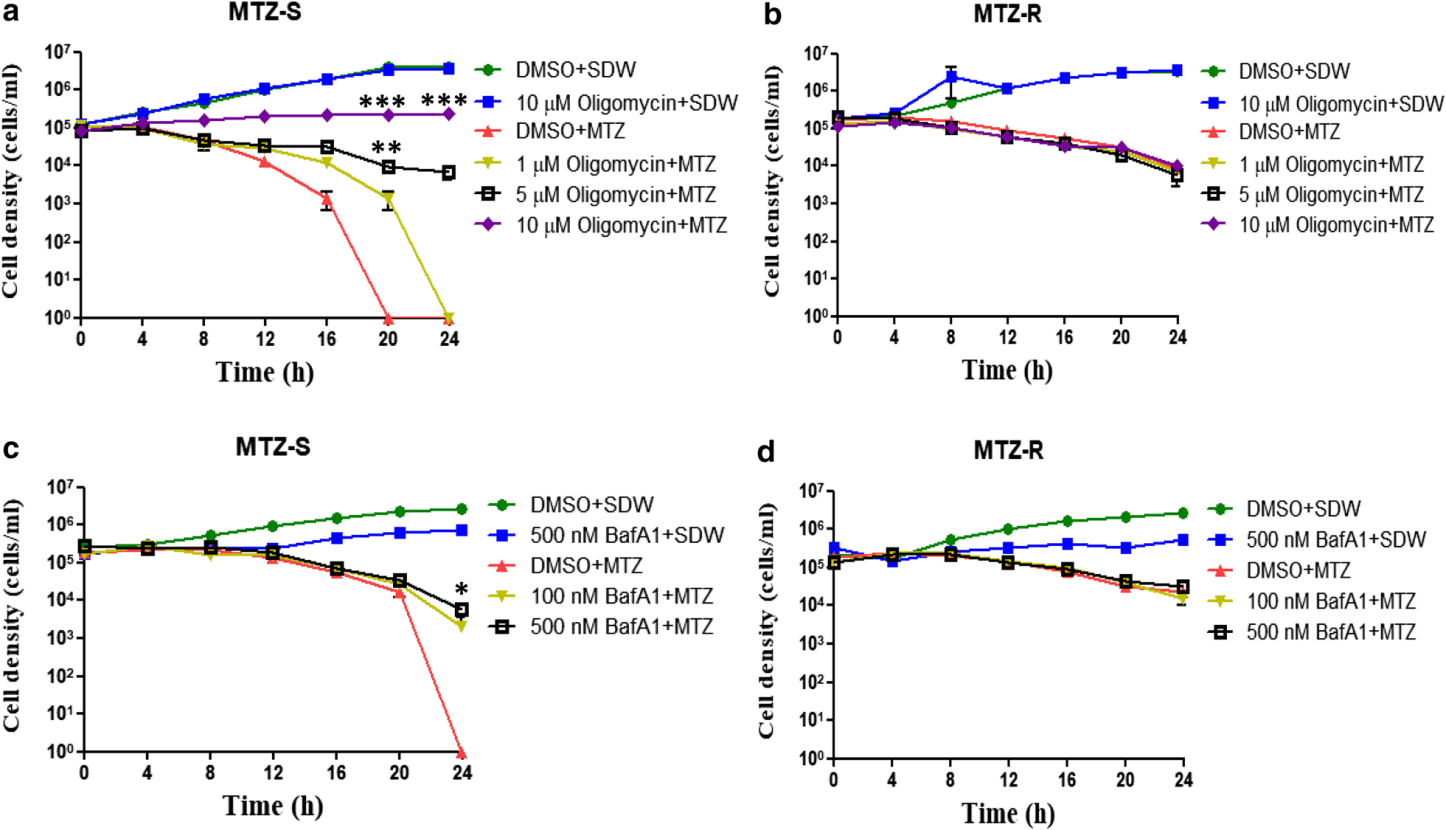
ATP synthases subunit-related proteins are associated with MTZ-resistance. The effects of MTZ (20 μM) on the growth of MTZ-S (**a**) and MTZ-R (**b**) parasites pretreated with different concentrations of oligomycin (1, 5 or 10 μM) were monitored. The effects of oligomycin (10 μM) on the growth of parasites were also monitored. Additionally, the effects of MTZ (20 μM) on the growth of MTZ-S (**c**) and MTZ-R (**d**) parasites pretreated with different concentrations of another ATP synthase inhibitor bafilomycin A1 (BafA1) (100 and 500 nM) were monitored. The effects of BafA1 (500 nM) on the growth of parasites were also monitored. Treatment with DMSO in combination with MTZ (20 μM) was served as the control to observe the effect of MTZ on the MTZ-S and MTZ-R parasites. The cell density was monitored every 4 h by trypan blue exclusion assay. The growth curves were presented as mean ± SD of three independent experiments. The statistical significances represent the effects of oligomycin and BafA1 on MTZ-treated parasites compared with the MTZ-treated control. *P < 0.05, **P < 0.01, ***P < 0.001

Oxidative phosphorylation was the most downregulated pathway in the MTZ-R proteome in response to drug treatment. It is noteworthy that most proteins categorized into the oxidative phosphorylation protein set were ATP synthase subunit-related proteins (Table 3), suggesting their potential roles in MTZ resistance. In the *T. vaginalis* genome, the genes encoding the subunits of putative V-type ATPase (A to H, a, c and d) have been identified [21], but their biological functions have not yet been investigated. Given that all the subunits of ATPase were significantly downregulated in the MTZ-R proteome in response to MTZ, we postulate that these ATP synthase-related proteins may sensitize trophozoites to drug treatment. We thus examined whether the ATP synthase inhibitors oligomycin and bafilomycin A1 (BafA1) are able to enhance the resistance of MTZ-S parasites to MTZ. The effects of MTZ on the growth of MTZ-S and MTZ-R parasites pretreated with different concentrations of oligomycin (1, 5 and 10 μM) (Fig. 6a, b) and BafA1 (100 nM and 500 nM) (Fig. 6c, d) were monitored. Notably, MTZ-S parasites pretreated with oligomycin and BafA1 enhanced the resistance to MTZ compared with the DMSO-treated control. However, the effects of ATP synthase inhibitors on the growth of MTZ-R following MTZ treatment were not observed. Together, these results suggest that inhibition of ATP synthase-related proteins may play a crucial role in MTZ resistance of *T. vaginalis.*

**Table 3:** ATP synthase subunit-related proteins in the oxidative phosphorylation protein set revealed by GSEA

Gene ID	Protein name	Ratio MTZ-R/MTZ-S
TVAG_292550	Vacuolar ATP synthase subunit f, putative	0.887
TVAG_262750	Vacuolar ATP synthase subunit H, putative	0.767
TVAG_021890	Vacuolar ATP synthase subunit E, putative	0.746
TVAG_438870	Vacuolar ATP synthase subunit E, putative	0.743
TVAG_420260	ATP synthase beta subunit, putative	0.708
TVAG_324980	ATP synthase, putative	0.687
TVAG_038640	ATP synthase subunit D, putative	0.604
TVAG_037610	Vacuolar ATP synthase subunit C, putative	0.594
TVAG_360810	Vacuolar ATP synthase subunit E, putative	0.523
TVAG_006020	Vacuolar ATP synthase subunit ac39, putative	0.492

## Discussion

MTZ resistance has been observed in 4.3–9.6% of clinical isolates of *T. vaginalis* in the USA [8, 39]; however, the underlying mechanism is far from understood. In the current study, we sought to advance our understanding of the global changes between the MTZ-R and MTZ-S proteomes in response to drug treatment, unraveling novel molecules or pathways associated with MTZ resistance. To our knowledge, this is the first quantitative proteomic analysis of MTZ resistance in trichomonads, showing many previously uncharacterized proteins with a potential impact on MTZ resistance.

A recent study demonstrated that the intracellular iron status is associated with early progression of MTZ resistance in *T. vaginalis*, but no significant changes in transcription of PFOR and Fd was observed between the MTZ-S and MTZ-R strains [40]. Additionally, no marked difference in PFOR mRNA levels is detected between the HM-1 and HM-1 derived MTZ-R strains of *E. histolytica* [20]. Our results showed inconsistent protein expression patterns of different isoforms of PFOR and Fd in the MTZ-R proteome. These findings suggest that multiple molecules or pathways are involved in MTZ resistance and that the underlying mechanism is regulated by different vaginal microenvironments. We found that all hydrogenosomal succinate STK family proteins are downregulated in the MTZ-R proteome after MTZ treatment, which is not consistent with the previous finding that shows almost the same protein expression levels of α-subunits of STK in the MTZ-S strain and its drug-resistant derivatives [18]. Hence, it is likely that various mechanisms of MTZ resistance exist in different isolates of *T. vaginalis*, which is similar to the recent study showing that the majority of differentially expressed proteins are unique to each of the three MTZ-R strains of *Giardia lamblia* [41]. Additionally, a transcriptomic study on MTZ resistance in tens of *T. vaginalis* strains [35] also demonstrates that their TrxR gene expression results are opposite to the expression pattern shown in previous studies [42].

Ankyrin 2,3/unc44 is the most upregulated protein in the MTZ-R proteome upon drug treatment, with about 30-fold higher expression compared to that in the MTZ-S proteome. It has been demonstrated that several bacterial and viral ankyrin repeat (ANK)-containing proteins play a role in host-pathogen interaction and the evolution of infectious diseases [43], but the biological significance of the ANK family proteins have not yet been well characterized in protists. A previous study reported that a gene homologous to ANKRD1 (ankyrin repeat domain 1[cardiac muscle]) is associated with the platinum sensitivity in ovarian cancer cell lines, and decreasing ANKRD1 expression is a potential strategy to sensitize tumors to platinum-based drugs [44]. Additionally, ankyrin binding to the multidrug transporter MDR1 (P-glycoprotein) results in the efflux of chemotherapeutic drugs in both human breast and ovarian tumor cells [45]. We have also identified two multidrug resistance proteins (TVAG_542450 and TVAG_542460) in the MTZ-R proteome after drug treatment (Additional file 2: Table S2). It is worth clarifying whether the identified MDR proteins are involved in MTZ resistance in *T. vaginalis*.

We found that several aminotransferases were upregulated in the MTZ-R proteome in response to MTZ. NAD-specific GDH, one of the aminotransferases that catalyzes the oxidative deamination of glutamate to α-ketoglutarate, has been shown to be upregulated as a part of the stress response in the MTZ-R strain of *E. histolytica* [20]. Our previous transcriptomic analysis in *T. vaginalis* also demonstrated the overexpression of aminotransferases upon glucose restriction, especially for GDH, which may serve as an energy compensation mechanism caused by glucose starvation [46]. Since we observed that the gene set of glycolysis was statistically downregulated and all the hydrogenosomal succinate STK family proteins that catalyze ATP synthesis were repressed in the MTZ-R proteome upon drug treatment, it is possible that the overexpression of aminotransferases also plays a compensatory role for energy production and parasite survival. It remains to be determined whether downregulation of hydrogenosomal STK in MTZ-R parasites upon MTZ exposure involves in the regulation of drug resistance in *T. vaginalis*.

Using GSEA, we have demonstrated that the oxidative phosphorylation protein set that contains ten ATP synthase subunit-related proteins was the most downregulated pathway in the MTZ-R proteome upon drug treatment. The drug resistance of MTZ-S parasites has been proved to be enhanced after pretreatment with two ATP synthase inhibitors oligomycin and bafilomycin A1, suggesting the potential association of these putative ATP synthases with drug susceptibility in *T. vaginalis*. ATP synthase is located on the membranes of mitochondria, bacteria, and chloroplast thylakoids as well as on the surfaces of various cell types, such as endothelial cells [47, 48], keratinocytes [49], and adipocytes [50]. It is noteworthy that downregulation of ATP synthase components has been reported in the majority of cancers [51, 52] and shown to be associated with chemotherapy resistance [53]. A recent study mechanistically demonstrated that loss of the ATP synthase subunit ATP5H is strongly linked to multimodal cancer therapy resistance and poor survival in cancer patients, and the mechanism is mediated by ROS accumulation and HIF-1 α stabilization that confers to tumor cells a stem-like and invasive phenotype [54]. A previous study in *Trypanosoma brucei* indicated that the V-ATPase subunit depleted strains display more than a 100-fold increase in the half-maximal effective concentration for the trypanocidal drug isometamidium chloride (ISM) [55]. Additionally, bafilomycin A1, a specific inhibitor of the V-ATPase that binds to the c subunit of V_0_, strongly antagonized ISM action. These findings conclude that V-ATPase sensitizes *T. brucei* to ISM. *Trichomonas vaginalis* lacks conventional mitochondria and instead contains divergent mitochondrial-related organelles called hydrogenosomes; however, the aforementioned ATP synthase subunit-related proteins have not been identified in the hydrogenosomal proteome [23, 26]. Some of the ATPase-related proteins have been identified by surface proteomic analysis of *T. vaginalis*, but their biological significance has not yet been verified. It is worthwhile to study which subunit of ATP synthase plays the most crucial role in MTZ resistance by gene-specific silencing. On the other hand, it will be also feasible to overexpress the specific ATP synthase in MTZ-R parasites and verify their susceptibility to MTZ. Together, our results suggest that loss of ATP synthase subunit-related proteins may be associated with MTZ resistance in *T. vaginalis*, with the potential to overcome drug resistance in trichomonads.

## Conclusions

Collectively, to the best of our knowledge, this study revealed for the first time the global proteomic changes between the MTZ-R and MTZ-S strains following drug treatment, enhancing our understanding of the molecular events potentially involved in MTZ resistance. We have highlighted the significantly enriched pathways as well as the specific proteins associated with MTZ resistance. Our results demonstrate that several aminotransferases categorized into amino acid metabolism were upregulated, whereas several proteins involved in glycolysis and hydrogenosomal energy metabolism were downregulated in the MTZ-R proteome in response to drug treatment. Specifically, ATP synthase subunit-related proteins classified into the oxidative phosphorylation gene set were the most downregulated family proteins in the MTZ-R proteome, and functional validation by ATP synthase inhibition assay supported the potential involvement of these proteins in MTZ susceptibility. We provide novel insights into the MTZ resistance mechanism at the proteome level, paving the way for future development of new drug targets against MTZ-refractory trichomoniasis.

## Supplementary information

**Additional file 1: Table S1.** Primers used in this study.

**Additional file 2: Table S2.** Differentially expressed proteins in the MTZ-R and MTZ-S proteomes treated with or without MTZ.

**Additional file 3: Figure S1.** Validation of the proteomic data by qPCR analysis. Four genes with 2-fold upregulation (receptor expression-enhancing protein and histidinol-phosphate aminotransferase) (**a**) and downregulation (glucose kinase and iron-sulfur flavoprotein) (**b**) in the MTZ-R proteome upon MTZ treatment compared with the MTZ-S proteome were confirmed by qPCR analysis. *P < 0.05, ** *P* < 0.01, *** *P* < 0.001.

**Additional file 4: Table S3.** Differentially expressed proteins involved in the hydrogenosomal energy metabolism in the MTZ-R proteome.

**Additional file 5: Table S4.** Enriched upregulated KEGG pathways in the MTZ-R proteome in response to MTZ treatment.

**Additional file 6: Table S5.** Enriched downregulated KEGG pathways in the MTZ-R proteome in response to MTZ treatment.

**Additional file 7: Table S6.** Enriched upregulated GO functional annotations in the MTZ-R proteome in response to MTZ treatment.

**Additional file 8: Table S7.** Enriched downregulated GO functional annotations in the MTZ-R proteome in response to MTZ treatment.

## Data Availability

Data supporting the conclusions of this article are included within the article and its additional files. The datasets used and/or analyzed during the present study will be made available by the corresponding author upon reasonable request. The mass spectrometry proteomics data have been deposited to the ProteomeXchange Consortium *via* PRIDE partner repository with the dataset identifier PXD018522.

## Abbreviations

MTZ: metronidazole
MTZ-S: MTZ-sensitive
MTZ-R: MTZ-resistant
PFOR: pyruvate:ferredoxin oxidoreductase
Fd: ferredoxin
STK: succinate thiokinase
GDH: glutamate dehydrogenase
ME: malic enzyme
ASCT: acetyl:succinate CoA-transferase
SDS-PAGE: sodium dodecyl sulfate polyacrylamide gel electrophoresis
BafA1: bafilomycin A1
