# Supplementary material for: Proteomic signatures of metronidazole-resistant Trichomonas vaginalis reveal novel proteins associated with drug resistance
Source: Parasit Vectors. 2020 Jun 1;13:274. doi: 10.1186/s13071-020-04148-5 (PMC7268490; doi:10.1186/s13071-020-04148-5)
Supplement: Supplementary file 1 — Additional file 1: Table S1. Primers used in this study. [file 13071_2020_4148_MOESM1_ESM.docx]

| **Additional file 1: Table S1 Primers used in this study** | | |
| --- | --- | --- |
| **Gene name** | **Locus** | **Primers** |
| Receptor expression-enhancing protein, putative | TVAG_232140 | F-5'-GGCTCCAACTTCCAGTATGC-3' |
|  |  | R-5'-AAGCGAAGCTCTGATTTCGT-3' |
| Histidinol-phosphate aminotransferase, putative | TVAG_108400 | F-5'-TCTTGGCACAGCACAAAGTC-3' |
|  |  | R-5'-GCGATACCACTTCCAACGAT-3' |
| Glucose kinase, putative | TVAG_442070 | F-5'-TCATGGCTGCTCGTGTAAAG-3' |
|  |  | R-5'-ATGATGCCACCACCGATAAT-3' |
| Iron-sulfur flavoprotein | TVAG_370510 | F-5'-CTGTTGGCAAAACAGCAGAA-3' |
|  |  | R-5'-TGCAAATGCGTCAACTTCAT-3' |
