# Supplementary figures and images for: Proteomic signatures of metronidazole-resistant Trichomonas vaginalis reveal novel proteins associated with drug resistance
Source: Parasit Vectors. 2020 Jun 1;13:274. doi: 10.1186/s13071-020-04148-5 (PMC7268490; doi:10.1186/s13071-020-04148-5)

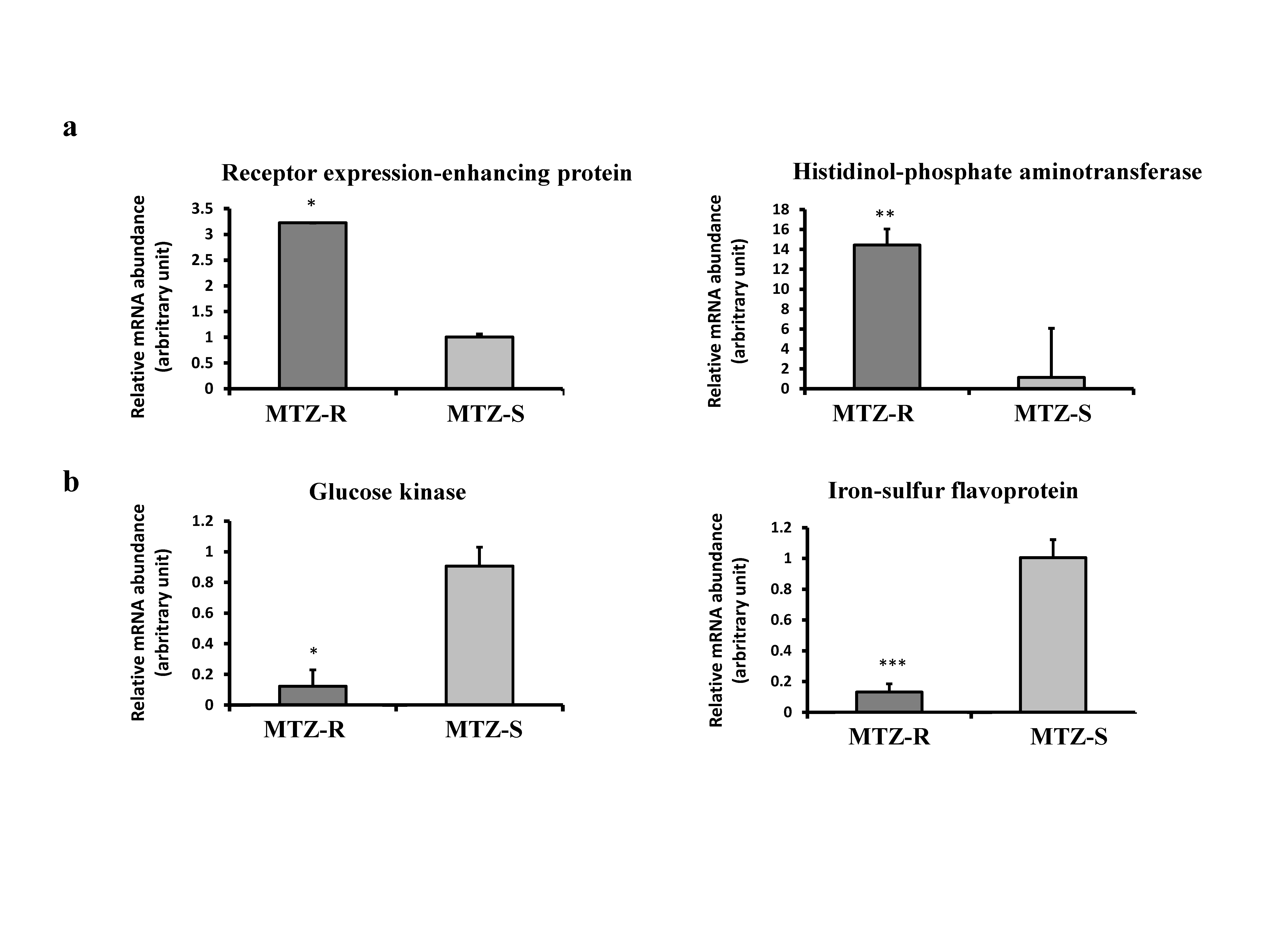

Supplement: Supplementary file 3 — Additional file 3: Figure S1. Validation of the proteomic data by qPCR analysis. Four genes with 2-fold upregulation (receptor expression-enhancing protein and histidinol-phosphate aminotransferase) (a) and downregulation (glucose kinase and iron-sulfur flavoprotein) (b) in the MTZ-R proteome upon MTZ treatment compared with the MTZ-S proteome were confirmed by qPCR analysis. *P < 0.05, ** P < 0.01, *** P < 0.001. [file 13071_2020_4148_MOESM3_ESM.tiff]
