# Supplementary material for: Proteomic signatures of metronidazole-resistant Trichomonas vaginalis reveal novel proteins associated with drug resistance
Source: Parasit Vectors. 2020 Jun 1;13:274. doi: 10.1186/s13071-020-04148-5 (PMC7268490; doi:10.1186/s13071-020-04148-5)
Supplement: Supplementary file 4 — Additional file 4: Table S3. Differentially expressed proteins involved in the hydrogenosomal energy metabolism in the MTZ-R proteome. [file 13071_2020_4148_MOESM4_ESM.docx]

| **Additional file 3: Table S3. Differentially expressed proteins involved in the hydrogenosomal energy metabolism in the MTZ-R proteome.** | | | |
| --- | --- | --- | --- |
| **Gene ID** | **TrichDB Annotation** | **MTZ-50143/MTZ-30236** | **50143/30236** |
| TVAG_164890 | Acetyl-CoA hydrolase | 1.58 | 1.79 |
| TVAG_395550 | Acetyl-CoA hydrolase | 0.95 | 1.10 |
| TVAG_117910 | alcohol dehydrogenase | 4.25 | 3.75 |
| TVAG_329660 | alcohol dehydrogenase | 1.45 | 1.51 |
| TVAG_329650 | alcohol dehydrogenase | 0.73 | 1.66 |
| TVAG_422780 | alcohol dehydrogenase | 0.70 | 0.67 |
| TVAG_001570 | alcohol dehydrogenase | 0.67 | 0.85 |
| TVAG_210250 | alcohol dehydrogenase | 0.44 | 1.05 |
| TVAG_228780 | alcohol dehydrogenase | 0.64 | 0.55 |
| TVAG_327470 | alcohol dehydrogenase | 1.29 | 1.15 |
| TVAG_328940 | alcohol dehydrogenase | 0.91 | 0.96 |
| TVAG_003900 | Ferredoxin 1 | 0.63 | 0.81 |
| TVAG_399860 | Ferredoxin 2 | 1.05 | 0.93 |
| TVAG_292710 | Ferredoxin 4 | 0.87 | 1.14 |
| TVAG_078730 | Ferredoxin 7 | 1.33 | 1.26 |
| TVAG_009460 | malic enzyme, putative | 1.90 | 2.64 |
| TVAG_320780 | malic enzyme, putative | 1.68 | 2.71 |
| TVAG_228520 | malic enzyme, putative | 1.42 | 2.18 |
| TVAG_491670 | malic enzyme, putative | 1.17 | 1.17 |
| TVAG_183790 | malic enzyme | 1.72 | 1.73 |
| TVAG_238830 | malic enzyme | 0.73 | 0.89 |
| TVAG_267870 | malic enzyme | 6.60 | 4.68 |
| TVAG_340290 | malic enzyme | 0.82 | 1.23 |
| TVAG_412220 | malic enzyme | 1.31 | 1.86 |
| TVAG_296220 | NADH dehydrogenase 24 kDa subunit | 0.69 | 0.97 |
| TVAG_072700 | NADH dehydrogenase 51 kDa subunit | 0.92 | 0.84 |
| TVAG_489800 | NADH dehydrogenase 51 kDa subunit | 1.43 | 1.07 |
| TVAG_037570 | NADH-ubiquinone oxidoreductase | 0.71 | 0.94 |
| TVAG_361590 | NADH-ubiquinone oxidoreductase | 1.44 | 1.00 |
| TVAG_133030 | NADH-ubiquinone oxidoreductase flavoprotein | 0.69 | 0.72 |
| TVAG_182620 | nitrate, fromate, iron dehydrogenase | 0.59 | 0.54 |
| TVAG_310050 | nitrate, fromate, iron dehydrogenase | 0.96 | 0.37 |
| TVAG_160930 | Periplasmic [Fe] hydrogenase | 1.68 | 0.97 |
| TVAG_096520 | pyruvate-flavodoxin oxidoreductase | 1.57 | 2.13 |
| TVAG_466790 | pyruvate-flavodoxin oxidoreductase | 2.22 | 3.23 |
| TVAG_198110 | pyruvate-flavodoxin oxidoreductase | 2.02 | 2.48 |
| TVAG_230580 | pyruvate-flavodoxin oxidoreductase | 0.89 | 1.18 |
| TVAG_242960 | pyruvate-flavodoxin oxidoreductase | 0.82 | 1.62 |
| TVAG_144730 | succinate thiokinase ? subunit | 0.93 | 0.81 |
| TVAG_183500 | succinate thiokinase ? subunit | 0.75 | 0.81 |
| TVAG_259190 | succinate thiokinase ? subunit | 0.58 | 0.94 |
| TVAG_047890 | succinate thiokinase a subunit | 0.49 | 0.81 |
| TVAG_318670 | succinate thiokinase a subunit | 0.60 | 1.14 |
