# Supplementary material for: Proteomic signatures of metronidazole-resistant Trichomonas vaginalis reveal novel proteins associated with drug resistance
Source: Parasit Vectors. 2020 Jun 1;13:274. doi: 10.1186/s13071-020-04148-5 (PMC7268490; doi:10.1186/s13071-020-04148-5)
Supplement: Supplementary file 5 — Additional file 5: Table S4. Enriched upregulated KEGG pathways in the MTZ-R proteome in response to MTZ treatment. [file 13071_2020_4148_MOESM5_ESM.docx]

| **Additional file 5: Table S4. Enriched upregulated KEGG pathways in the MTZ-R proteome in response to MTZ treatment** | | | |
| --- | --- | --- | --- |
| **GS^a^** | **SIZE^b^** | **ES^c^** | **NES^d^** |
| ARGININE AND PROLINE METABOLISM | 14 | 0.66 | 1.71 |
| ALANINE, ASPARTATE AND GLUTAMATE METABOLISM | 12 | 0.65 | 1.65 |
| RIBOSOME BIOGENESIS IN EUKARYOTES | 43 | 0.44 | 1.44 |
| PEROXISOME | 10 | 0.61 | 1.44 |
| PURINE METABOLISM | 36 | 0.42 | 1.34 |
| STARCH AND SUCROSE METABOLISM | 17 | 0.46 | 1.24 |
| CYSTEINE AND METHIONINE METABOLISM | 11 | 0.49 | 1.18 |
| MRNA SURVEILLANCE PATHWAY | 26 | 0.38 | 1.15 |
| RNA TRANSPORT | 34 | 0.36 | 1.13 |
| PENTOSE PHOSPHATE PATHWAY | 18 | 0.41 | 1.13 |
| RNA POLYMERASE | 14 | 0.43 | 1.12 |
| PYRIMIDINE METABOLISM | 30 | 0.34 | 1.04 |
| DNA REPLICATION | 19 | 0.36 | 0.98 |
| RNA DEGRADATION | 15 | 0.37 | 0.97 |
| NUCLEOTIDE EXCISION REPAIR | 14 | 0.33 | 0.85 |
| MISMATCH REPAIR | 13 | 0.32 | 0.83 |
| PYRUVATE METABOLISM | 16 | 0.31 | 0.83 |
| PROTEIN PROCESSING IN ENDOPLASMIC RETICULUM | 28 | 0.27 | 0.81 |
| AMINOACYL-TRNA BIOSYNTHESIS | 20 | 0.28 | 0.80 |
| RIBOSOME | 49 | 0.23 | 0.76 |
| UBIQUITIN MEDIATED PROTEOLYSIS | 15 | 0.27 | 0.69 |
| ^a^ Gene set name. ^b^ Number of genes in the gene set. ^c^ Enrichment score for the gene set, which reflects the degree to which the gene set is overrepresented at the top or bottom of the ranked list of genes. ^d^ The ES for the gene set that has been normalized across analyzed gene sets. | | | |
