# Supplementary material for: Proteomic signatures of metronidazole-resistant Trichomonas vaginalis reveal novel proteins associated with drug resistance
Source: Parasit Vectors. 2020 Jun 1;13:274. doi: 10.1186/s13071-020-04148-5 (PMC7268490; doi:10.1186/s13071-020-04148-5)
Supplement: Supplementary file 6 — Additional file 6: Table S5. Enriched downregulated KEGG pathways in the MTZ-R proteome in response to MTZ treatment. [file 13071_2020_4148_MOESM6_ESM.docx]

| **Additional file 6: Table S5. Enriched downregulated KEGG pathways in the MTZ-R proteome in response to MTZ treatment** | | | |
| --- | --- | --- | --- |
| **GS^a^** | **SIZE^b^** | **ES^c^** | **NES^d^** |
| OXIDATIVE PHOSPHORYLATION | 13 | -0.89 | -2.83 |
| CITRATE CYCLE (TCA CYCLE) | 13 | -0.58 | -1.73 |
| GLYCOLYSIS / GLUCONEOGENESIS | 23 | -0.45 | -1.73 |
| PHAGOSOME | 22 | -0.43 | -1.60 |
| GALACTOSE METABOLISM | 11 | -0.52 | -1.46 |
| FRUCTOSE AND MANNOSE METABOLISM | 12 | -0.44 | -1.33 |
| AMINO SUGAR AND NUCLEOTIDE SUGAR METABOLISM | 18 | -0.34 | -1.15 |
| ENDOCYTOSIS | 16 | -0.22 | -0.73 |
| PROTEIN EXPORT | 10 | -0.23 | -0.64 |
| PROTEASOME | 37 | -0.15 | -0.61 |
| ^a^ Gene set name. ^b^ Number of genes in the gene set. ^c^ Enrichment score for the gene set, which reflects the degree to which the gene set is overrepresented at the top or bottom of the ranked list of genes. ^d^ The ES for the gene set that has been normalized across analyzed gene sets. | | | |
