# Supplementary material for: Proteomic signatures of metronidazole-resistant Trichomonas vaginalis reveal novel proteins associated with drug resistance
Source: Parasit Vectors. 2020 Jun 1;13:274. doi: 10.1186/s13071-020-04148-5 (PMC7268490; doi:10.1186/s13071-020-04148-5)
Supplement: Supplementary file 7 — Additional file 7: Table S6. Enriched upregulated GO functional annotations in the MTZ-R proteome in response to MTZ treatment. [file 13071_2020_4148_MOESM7_ESM.docx]

| **Additional file 7: Table S6. Enriched upregulated GO functional annotations in the MTZ-R proteome in response to MTZ treatment** | | | |
| --- | --- | --- | --- |
| **GS^a^** | **SIZE^b^** | **ES^c^** | **NES^d^** |
| MOLECULAR FUNCTION ACTIN BINDING | 19 | 0.63 | 1.70 |
| MOLECULAR FUNCTION CYSTEINE TYPE PEPTIDASE ACTIVITY | 15 | 0.61 | 1.61 |
| CELLULAR COMPONENT TUBULIN COMPLEX | 17 | 0.59 | 1.61 |
| BIOLOGICAL PROCESS RESPONSE TO HEAT | 13 | 0.58 | 1.47 |
| MOLECULAR FUNCTION 2 ALKENAL REDUCTASE NAD P ACTIVITY | 14 | 0.57 | 1.46 |
| BIOLOGICAL PROCESS GLYCEROLIPID METABOLIC PROCESS | 22 | 0.49 | 1.44 |
| BIOLOGICAL PROCESS PROTEOLYSIS | 60 | 0.42 | 1.43 |
| CELLULAR COMPONENT CHROMATIN | 14 | 0.54 | 1.39 |
| BIOLOGICAL PROCESS REGULATION OF GTPASE ACTIVITY | 14 | 0.54 | 1.38 |
| CELLULAR COMPONENT MYOSIN COMPLEX | 15 | 0.52 | 1.38 |
| BIOLOGICAL PROCESS MICROTUBULE BASED MOVEMENT | 10 | 0.56 | 1.34 |
| MOLECULAR FUNCTION ZINC ION BINDING | 54 | 0.38 | 1.28 |
| MOLECULAR FUNCTION PHOSPHOLIPID BINDING | 10 | 0.53 | 1.26 |
| BIOLOGICAL PROCESS STREPTOMYCIN BIOSYNTHETIC PROCESS | 13 | 0.50 | 1.26 |
| BIOLOGICAL PROCESS SIGNAL TRANSDUCTION | 13 | 0.50 | 1.26 |
| CELLULAR COMPONENT ENDOPLASMIC RETICULUM | 20 | 0.45 | 1.25 |
| BIOLOGICAL PROCESS SERINE FAMILY AMINO ACID METABOLIC PROCESS | 88 | 0.34 | 1.25 |
| MOLECULAR FUNCTION RNA BINDING | 12 | 0.49 | 1.23 |
| MOLECULAR FUNCTION HYDROLASE ACTIVITY | 27 | 0.41 | 1.21 |
| MOLECULAR FUNCTION PROTEIN HOMODIMERIZATION ACTIVITY | 10 | 0.50 | 1.20 |
| CELLULAR COMPONENT MITOCHONDRION | 27 | 0.40 | 1.19 |
| MOLECULAR FUNCTION NUCLEOSIDE TRIPHOSPHATASE ACTIVITY | 10 | 0.50 | 1.19 |
| MOLECULAR FUNCTION UBIQUITIN PROTEIN LIGASE ACTIVITY | 16 | 0.44 | 1.19 |
| BIOLOGICAL PROCESS OXIDATION REDUCTION PROCESS | 52 | 0.34 | 1.18 |
| CELLULAR COMPONENT RIBOSOME | 83 | 0.32 | 1.17 |
| CELLULAR COMPONENT CYTOSOL | 21 | 0.41 | 1.16 |
| MOLECULAR FUNCTION TRANSLATION INITIATION FACTOR ACTIVITY | 13 | 0.45 | 1.15 |
| BIOLOGICAL PROCESS SUCROSE METABOLIC PROCESS | 28 | 0.37 | 1.13 |
| CELLULAR COMPONENT PROTEASOME COMPLEX | 19 | 0.40 | 1.11 |
| BIOLOGICAL PROCESS UBIQUITIN DEPENDENT PROTEIN CATABOLIC PROCESS | 11 | 0.45 | 1.10 |
| MOLECULAR FUNCTION DNA BINDING | 13 | 0.43 | 1.10 |
| MOLECULAR FUNCTION OXIDOREDUCTASE ACTIVITY | 12 | 0.43 | 1.06 |
| CELLULAR COMPONENT MEMBRANE | 49 | 0.32 | 1.06 |
| BIOLOGICAL PROCESS PENTOSE PHOSPHATE SHUNT | 20 | 0.37 | 1.05 |
| BIOLOGICAL PROCESS PROTEIN DEUBIQUITINATION | 10 | 0.44 | 1.04 |
| MOLECULAR FUNCTION GTP BINDING | 96 | 0.28 | 1.03 |
| MOLECULAR FUNCTION GTPASE ACTIVITY | 44 | 0.31 | 1.03 |
| MOLECULAR FUNCTION CALCIUM ION BINDING | 32 | 0.33 | 1.01 |
| BIOLOGICAL PROCESS PROTEIN UBIQUITINATION | 16 | 0.38 | 1.01 |
| BIOLOGICAL PROCESS CARBON UTILIZATION | 32 | 0.32 | 1.01 |
| CELLULAR COMPONENT NUCLEUS | 114 | 0.27 | 1.00 |
| MOLECULAR FUNCTION PROTEIN SERINE THREONINE KINASE ACTIVITY | 32 | 0.33 | 1.00 |
| BIOLOGICAL PROCESS THREONINE METABOLIC PROCESS | 13 | 0.39 | 0.99 |
| CELLULAR COMPONENT GOLGI APPARATUS | 17 | 0.36 | 0.99 |
| MOLECULAR FUNCTION PROTEIN SERINE THREONINE PHOSPHATASE ACTIVITY | 10 | 0.42 | 0.99 |
| BIOLOGICAL PROCESS REGULATION OF TRANSLATIONAL INITIATION | 30 | 0.32 | 0.98 |
| MOLECULAR FUNCTION PHOSPHOPROTEIN PHOSPHATASE ACTIVITY | 10 | 0.41 | 0.98 |
| CELLULAR COMPONENT TRANSCRIPTION FACTOR COMPLEX | 22 | 0.34 | 0.98 |
| CELLULAR COMPONENT CYTOPLASM | 119 | 0.25 | 0.97 |
| MOLECULAR FUNCTION UNFOLDED PROTEIN BINDING | 35 | 0.30 | 0.95 |
| MOLECULAR FUNCTION MAGNESIUM ION BINDING | 16 | 0.36 | 0.95 |
| BIOLOGICAL PROCESS PROTEIN DEPHOSPHORYLATION | 17 | 0.35 | 0.94 |
| BIOLOGICAL PROCESS GLYOXYLATE METABOLIC PROCESS | 11 | 0.39 | 0.93 |
| BIOLOGICAL PROCESS RRNA PROCESSING | 11 | 0.38 | 0.93 |
| MOLECULAR FUNCTION STRUCTURAL MOLECULE ACTIVITY | 20 | 0.33 | 0.92 |
| BIOLOGICAL PROCESS GTP CATABOLIC PROCESS | 10 | 0.38 | 0.91 |
| MOLECULAR FUNCTION CYSTEINE TYPE ENDOPEPTIDASE ACTIVITY | 10 | 0.38 | 0.90 |
| BIOLOGICAL PROCESS PROTEIN TRANSPORT | 56 | 0.26 | 0.87 |
| CELLULAR COMPONENT INTRACELLULAR | 37 | 0.27 | 0.86 |
| CELLULAR COMPONENT NUCLEOLUS | 27 | 0.28 | 0.85 |
| CELLULAR COMPONENT PLASMA MEMBRANE | 71 | 0.24 | 0.84 |
| BIOLOGICAL PROCESS RIBOSOME BIOGENESIS | 78 | 0.23 | 0.84 |
| BIOLOGICAL PROCESS PYRIMIDINE BASE METABOLIC PROCESS | 37 | 0.25 | 0.79 |
| MOLECULAR FUNCTION NUCLEOTIDE BINDING | 28 | 0.26 | 0.79 |
| MOLECULAR FUNCTION METAL ION BINDING | 11 | 0.32 | 0.78 |
| BIOLOGICAL PROCESS PHOSPHORYLATION | 13 | 0.30 | 0.77 |
| MOLECULAR FUNCTION STRUCTURAL CONSTITUENT OF RIBOSOME | 51 | 0.23 | 0.77 |
| BIOLOGICAL PROCESS PROTEIN FOLDING | 13 | 0.31 | 0.77 |
| MOLECULAR FUNCTION ATP BINDING | 199 | 0.19 | 0.75 |
| BIOLOGICAL PROCESS RESPONSE TO STRESS | 13 | 0.25 | 0.64 |
| CELLULAR COMPONENT INTEGRAL TO MEMBRANE | 32 | 0.21 | 0.63 |
| BIOLOGICAL PROCESS ELECTRON TRANSPORT | 21 | 0.22 | 0.63 |
| BIOLOGICAL PROCESS ACYL CARRIER PROTEIN BIOSYNTHETIC PROCESS | 19 | 0.22 | 0.62 |
| MOLECULAR FUNCTION PROTEIN TRANSPORTER ACTIVITY | 28 | 0.21 | 0.62 |
| BIOLOGICAL PROCESS GLYCOSPHINGOLIPID METABOLIC PROCESS | 47 | 0.17 | 0.57 |
| MOLECULAR FUNCTION SPHINGOMYELIN PHOSPHODIESTERASE ACTIVITY | 20 | 0.16 | 0.44 |
| MOLECULAR FUNCTION METHYLTRANSFERASE ACTIVITY | 12 | 0.16 | 0.39 |
| MOLECULAR FUNCTION TRANSFERASE ACTIVITY | 10 | 0.14 | 0.34 |
| ^a^ Gene set name. ^b^ Number of genes in the gene set. ^c^ Enrichment score for the gene set, which reflects the degree to which the gene set is overrepresented at the top or bottom of the ranked list of genes. ^d^ The ES for the gene set that has been normalized across analyzed gene sets. | | | |
