# Supplementary material for: Proteomic signatures of metronidazole-resistant Trichomonas vaginalis reveal novel proteins associated with drug resistance
Source: Parasit Vectors. 2020 Jun 1;13:274. doi: 10.1186/s13071-020-04148-5 (PMC7268490; doi:10.1186/s13071-020-04148-5)
Supplement: Supplementary file 8 — Additional file 8: Table S7. Enriched downregulated GO functional annotations in the MTZ-R proteome in response to MTZ treatment. [file 13071_2020_4148_MOESM8_ESM.docx]

| **Additional file 8: Table S7. Enriched downregulated GO functional annotations in the MTZ-R proteome in response to MTZ treatment** | | | |
| --- | --- | --- | --- |
| **GS^a^** | **SIZE^b^** | **ES^c^** | **NES^d^** |
| MOLECULAR FUNCTION PROTEIN DISULFIDE OXIDOREDUCTASE ACTIVITY | 11 | -0.52 | -1.51 |
| MOLECULAR FUNCTION THREONINE TYPE ENDOPEPTIDASE ACTIVITY | 14 | -0.43 | -1.40 |
| BIOLOGICAL PROCESS GLYCOLYSIS | 10 | -0.43 | -1.25 |
| MOLECULAR FUNCTION AMINOPEPTIDASE ACTIVITY | 12 | -0.38 | -1.19 |
| CELLULAR COMPONENT CLATHRIN ADAPTOR COMPLEX | 21 | -0.31 | -1.16 |
| BIOLOGICAL PROCESS PROLINE METABOLIC PROCESS | 12 | -0.26 | -0.81 |
| BIOLOGICAL PROCESS INTRACELLULAR PROTEIN TRANSPORT | 59 | -0.15 | -0.67 |
| BIOLOGICAL PROCESS L PHENYLALANINE BIOSYNTHETIC PROCESS | 14 | -0.21 | -0.66 |
| BIOLOGICAL PROCESS METABOLIC PROCESS | 16 | -0.19 | -0.65 |
| CELLULAR COMPONENT CHLOROPLAST | 15 | -0.19 | -0.61 |
| CELLULAR COMPONENT MEMBRANE COAT | 11 | -0.20 | -0.57 |
| ^a^ Gene set name. ^b^ Number of genes in the gene set. ^c^ Enrichment score for the gene set, which reflects the degree to which the gene set is overrepresented at the top or bottom of the ranked list of genes. ^d^ The ES for the gene set that has been normalized across analyzed gene sets. | | | |
